# The Medical Portrait Gallery

**Published:** 1839

**Authors:** 


### THE MEDICAL PORTRAIT GALLERY.

BIOGRAPHICAL MEMOIRS OF THE MOST CELEBRATED PHYSICIANS, SURGEONS, &c. &c. &c. By *Thomas Joseph Pettigrew*, F.R.S. &c. &c. &c. Parts XIV. and XV. Price 3s. each Part.

OUR readers will perceive that, in spite of positive obstacles and of more discouraging apathy, Mr. Pettigrew still continues this work. He has changed his publisher, and partially altered his plan, arrangements from which he expects, and seems not unlikely to derive advantage.

It is both a natural and a beneficial feeling, which induces us to pry into the lives of illustrious men—to observe the more secret springs of action, the elements of success, and the qualities of mind which have contributed to make them famous. If such curiosity reduces the intellectual giant to every-day proportions, it encourages the man of such proportions to imitate the conduct and attain the dimensions of the giant. It teaches us that however great the end, the means have been usually homely and practicable by almost all. It breeds emulation because it brings its fruits within the grasp of sober reality, and, stripping success of the mist which distance and imperfect knowledge throw around it, we perceive that, with determination, we ourselves may seize it.

In a profession like our's, torn by dissensions, distracted by jealousies, some common standards of professional excellence become peculiarly requisite. They exercise a powerful influence on our minds, manners, and conduct. We are not only told that such is professional honour or such professional merit, but we see that they have led, and we feel that they must lead again, to professional success. These breathing examples silence casuistry, convince scepticism. They shew that no man is permanently fortunate and happy in medicine, who has not high deserts—and those, more particularly, industry and rectitude. Lord Byron never said anything truer than that—"nothing but virtue will do, after all, in this d——d world."

We are inclined to notice these medical biographies more fully than we have done. If we diffuse harmony, if we spread zeal, if we disseminate and nurture emulation, we do as great a service to the profession, as we should do by communicating a fact in medicine. Probably we do a greater service in the former than the latter case, for a right spirit excited may lead to great things.

The first of the parts before us contains a memoir and a portrait of Dr. Heberden, and of Dr. Philip. We shall not go elaborately into the career of each Worthy as we cite him; but content ourselves with offering a few particulars, interesting to us and characteristic of him. And first of Dr. HEBERDEN.

Dr. William Heberden, the son of Richard Heberden, was born in London, in the year 1710. On the 17th of June, 1717, he was admitted into the Grammar School of St. Saviour, where he acquired the rudiments of his education. In 1724, he entered at St. John's College, Cambridge; and took the degree of B.A. in 1728, and of M.A. in 1732. He obtained a fellowship in 1730, and then directed his attention to the study of medicine. Having taken the degree of M.D. in 1739, he practised his profession in the University for ten years. He read an annual course of Lectures on the Materia Medica; and the specimens collected by him for this purpose were, in 1750, presented by him to his College.

"Among his pupils were many, who, in after life, honourably distinguished themselves; of whom it is sufficient to mention, Sir George Baker, Bart., Physician to their Majesties, Dr. Gisborne, President of the College of Physicians in 1797, and Dr. Robert Glynn, well known by his eccentricities."*

In 1746 Dr. Heberden was elected a Fellow of the Royal College of Physicians, and two years afterwards he settled in London, where he rapidly attained to great professional reputation. He would have taken this step at an earlier period, but for some juggling, the nature of which does not clearly come out. On commencing practice in the metropolis, he fixed his residence in Cecil Street. In 1749, he was chosen a Fellow of the Royal Society; and to the Philosophical Transactions, he was a contributor of four papers:—1, An account of a very large human calculus; 2, An account of the effects of lightning at South Weald, in Essex; 3, An account of a stone voided without help from the bladder of a woman, at Bury; 4, Of the different quantities of rain which appear to fall, at different heights, over the same spot of ground.

In 1760, Dr. Heberden married Mary, the eldest daughter of William Wollaston, Esq., by whom he had eight children, two only of whom survived

---

* "Dr. Glynn was educated at Eton, and went to King's College. He obtained the Seatonian Prize at Cambridge, in 1757, the subject being a Poem on 'The Day of Judgment.' He was well known for his wit and humour, for the good qualities of his heart, for his benevolence, and for his learning. He lived to the age of 82, and died in 1800. His physiognomy had a peculiar expression of archness and was unprepossessing, and there is a *jeu d'esprit* recorded, which seems to allude to this circumstance:

'This morning, quite dead, Tom was found in his bed,

Although he was hearty last night;

But 'tis thought, having seen Dr. Glynn in a dream,

That the poor fellow died of the fright.'"

him, the present Dr. William Heberden, and Mary, who married the Rev. G. Genyns, Prebendary of Ely.

Dr. Heberden died in Pall Mall on the 17th of May, 1801, at which time he had reached his 91st year, and was the senior fellow of the College of Physicians. The serenity of his mind at this trying hour is shown by the recorded fact, that within forty-eight hours of his decease, he repeated a sentence from an ancient Roman author, signifying that "Death is kinder to none than those to whom he comes uninvoked."

In 1796, he met with an accident that disabled him for the remainder of his life. Previous to this he had been in the habit of taking daily exercise. His cheerfulness was in no degree disturbed by this occurrence, for when he was fast approaching to the age of 90, he observed that though his occupations and pleasures were certainly changed from what they had used to be, yet he knew not if he had ever passed a year more comfortably than the last.

It must be stated, to Dr. Heberden's honour, that he retired from practice many years before his death—and the reason why he did so is creditable to his sagacity. He withdrew, in a great measure, at the age of 72. His resolution to retire seems to have been formed in consequence of the impairment of the faculties of Dr. Mead, which Dr. Heberden witnessed in a consultation on the Duke of Leeds. It is reported that Dr. H. then determined within himself, that if ever he lived to the same age (78) he would give up practice. In a letter to Dr. Perceval, written in 1794, he thus alludes to his retirement:—

"I have entered my 85th year; and when I retired, a few years ago, from the practice of physic, I trust it was not from a wish to be idle, which no man capable of being usefully employed, has a right to be; but because I was willing to give over, before my presence of thought, judgment, and recollection was so impaired, that I could not do justice to my patients. It is more desirable for a man to do this a little too soon, than a little too late; for the chief danger is on the side of not doing it soon enough." 6.

How true is this, and how high must be our estimate of the good sense of a man, who could not only form such correct opinions, but *act* on them! All see, and deplore the ravages of time on others—few willingly admit them in their own persons. And yet it is sad to see men who have outlived their faculties and reputations, still struggling for practice, and descending from the high position of giving the law, to suffer the opposition of contempt, or the humiliating deference of pity.

It is much to be regretted that there are not situations of dignified retirement for the older members of our profession—situations like the judgeships in law, which might serve as a stage between active practice and absolute withdrawal from it. Were there such situations, we should not witness such painful spectacles as few amongst us but have seen—the public would not be exposed to reputation without the faculties which gave it birth—and the younger talent amongst us would find a clearer stage to play its part. There are not such situations: but there is a monitor in every man's bosom which should warn him, and will, if he is disposed to listen, that mental, like physical strength, decays; but that, unlike physical, its weakness does not necessarily carry with it the consciousness of its existence. The octogenarian feels that he cannot run a race—but he often does not know that his is the garrulity of dotage. No strength of faculty, no force of reason is proof against the stern law of organization and of nature; and it is while the faculties are strong and reason vigorous, that the resolution should be firmly taken, to withdraw from active life while the intellect continues unimpaired. Who, of a noble or a generous nature, could bear to contemplate himself an object of compassion, "a driveller and a show," in scenes and amidst men where he once shone as a master mind?

Dr. Heberden possessed classical attainments of a high order, and he was liberal in his patronage of works of erudition and of merit. Such a taste would naturally tend to intimacy with literary and scientific men; and Gray, Bishop Hurd, Bishop Lowth, Cavendish, Dr. Jortin, were a few among his associates of this character.

His mind seems to have been naturally inclined to piety, and in the lineaments of his grave and benevolent countenance, a sense of religion is strongly marked. His conduct coincided with this apparent bias of his temperament, and the following anecdote will raise his character in the estimation of the Christian reader.

"Understanding that Dr. C. Middleton had composed a book on the 'Inefficacy of Prayer,' Dr. H. called upon his widow soon after the Dr.'s death, and asked her if she was not in possession of such a tract? She answered that she was; he then asked her if any bookseller had been in treaty with her for it? She said that a bookseller had offered her £50. for it. He then demanded if there was a duplicate? 'No;' upon that he requested to see it, and she immediately brought it, and put it into his hands. The Dr. holding it in one hand, and giving it a slight perusal, threw it into the fire, and with the other hand gave her a £50. note."

In Dr. Heberden the practical accompanied the speculative religious feeling, and whilst his faith was lively, his works would have done honour to any creed. His charity to the poor was more extended even than his protection of literary men. There was scarcely a public institution to which he did not liberally subscribe, and his private donations were numerous and large. As soon as his professional emoluments were adequate to his wants, he resigned the fellowship of St. John's, that some poorer scholar might be benefitted by the appointment. "By qualities of this kind," says a slight biographer, "accompanied with great sweetness of manners, he acquired the love and esteem of all good men, in a degree which, perhaps, very few have experienced; and, after passing an active life, with the uniform testimony of a good conscience, he became an eminent example of its influence, in the cheerfulness and serenity of his latest age."

Dr. Wells addressed Lord Kenyon, and will please any reader of taste, with a sketch of Dr. Heberden scarcely more felicitous in style, than correct in fact. Dr. Wells diffidently supposes himself engaged in the attempt to describe Dr. Heberden's character.

"I should first mark his various and extensive learning, his modesty in the use of it, and philosophical distrust of human opinions in science, however sanctioned by time, or the authority of great names. I should then exhibit him in the exercise of his profession, without envy or jealousy; too proud to court employment, yet underrating his services after they were performed; unwearied, even when a veteran in his art, in ascertaining the minutest circumstances of the sick, who placed themselves under his care, taking nothing in their situation for granted, that might be learned by inquiry, and trusting nothing of importance that concerned them to his memory. To demonstrate his greatness of mind, I should next mention his repeatedly declining to accept those offices of honour and profit at the British Court, which are regarded by other physicians as objects of their highest ambition, and are therefore sought by them with the utmost assiduity. I should afterwards take notice of his simple, yet dignified manners, his piety to God, his love for his country, and his exemplary discharge of the duties of all the private relations in which he stood to society; and I should conclude, by observing, that his whole life had been regulated by the most exquisite prudence, by means of which his other virtues were rendered more conspicuous and useful; and whatever failings, he might as a human being possess, were either shaded or altogether concealed. After my description was finished, I should think it proper to say, that I had never been acquainted with Dr. Heberden, and consequently could neither be dazzled by the splendour of his virtues, from approaching them too nearly, nor influenced in my opinion concerning them, by benefits he had already conferred upon me; and that standing, as he does, upon the verge of this state of existence, ready to wing his flight to another of glory, his ear must now be closed to the voice of flattery, had he ever listened to that syren, or were I base enough to solicit her aid, in the foolish expectation of receiving from him some future reward." 10.

A noble tribute to departing worth, a tribute drawn forth by Dr. Wells's opposition to that Old College of Physicians, of whose fellows Lord Kenyon supposed with great simplicity, Dr. Heberden a sample! He adorned the College, but he did not represent it. The spirit of bigotry, intolerance, and uncharitableness, which found its incarnation in that *old* Corporation, must be represented by other instruments than Dr. Heberden.

Dr. Heberden's earliest published work was a little pamphlet, entitled, ANTIΘΗΡΙΑΚΑ, in the form of *An Essay on Mithridatium and Theriaca.*

" The famous antidote to the baneful effects of poisons said to be possessed by Mithridates, King of Pontus, is here shewn to have been composed of the most simple ingredients: namely, twenty leaves of rue, one grain of salt, two nuts, and two dried figs. This is given upon the authority of Q. Serenus Samonicus, and is a very different composition to that named after the monarch, in the time of Celsus, which consisted of thirty-eight simples, and these it appears, were changed every century; so that the Mithridatium of one period bore little or no resemblance to that of another. Dr. Heberden states, that the ancients were only acquainted with *three* kinds of poison—hemlock, aconite, and that of venomous beasts, and to these they knew no antidotes. The various effects of different substances may, probably, in the earlier ages, have excited the apprehensions of mankind, and their ignorance of their nature may have given rise to the relation of miraculous stories regarding poisonous substances. Dr. H. very properly ridicules these accounts; such as the concealing of poisons under the stone of a seal or ring, as reported by Theophrastus, or by vapours arising from perfumed gloves or letters. Many modes of poisoning are mentioned, as having been practised by the Italians, among others that of poisoning the clothes. Ben Jonson, in his ' Every Man in his Humour,' puts the following passage into the mouth of the jealous Kitely:

' Now, God forbid, O me, now I remember,
My wife drank to me last, and changed the cup;
And bade me wear this *cursed suit* to-day.'

These practices were not confined to Italy. Our own country shared the like, and we learn that Queen Elizabeth was in constant dread of being taken off in this way. Two men were hanged in 1598, for poisoning her saddle!" 10.

The Transactions of the Royal College of Physicians, known by the title of "Medical Transactions," were undertaken at the recommendation of Dr. Heberden. He contributed not only largely to the work, but some of the most valuable papers in it are his. It would seem that Dr. Heberden had *intended* drawing up a copious prefatory address. A sketch of that address remains, and its object was to recommend the study of nature, in place of the deference to authority which characterized the physicians of his and of former days. The following passage will be read with double interest:—because it contains some great and admirable truths—and because it shows that Dr. Heberden's mind was amply fortified with the bold spirit of the Inductive Philosophy.

"The deference which is sometimes required in physic to the authority of the ancients, would incline any one to suspect, that the improvements in the art of healing have not kept pace with those which have been made in other branches of natural knowledge. Philosophers have long ago thrown off Aristotle's tyranny; yet some physicians still choose to wrangle about the meaning of the ancients, rather than to consult nature herself. Are they afraid of approaching her immediate presence, without making use of the intercession of Hippocrates and Galen? And is that reverence to be still paid to her once faithful ministers, which is properly due to nature alone, notwithstanding all that Bacon, and Harvey, and Newton, and our other great reformers, have witnessed against this mistaken veneration? In works of genius the ancients are unquestionably our superiors and best patterns; but in that sort of knowledge which depends wholly upon experience, the latest writers must in general be the best.—Experience may, in politics and morality, be called the teacher of fools; but in the study of nature, there is no other guide to true knowledge: accordingly, the practice of physic has been more improved by the casual experiments of illiterate nations, and the rash ones of vagabond quacks, than by the reasonings of all the once celebrated professors of it, and theoretic teachers in the several schools of Europe: very few of whom have furnished us with one new medicine, or have taught us better to use our old ones, or have in any one instance at all improved the art of curing diseases." 12.

Mr. Pettigrew adds:—

"This opinion accords well with that given by Sir William Temple who says, 'I had ever quarrelled with their studying art more than nature, and applying themselves to methods rather than to remedies; whereas the knowledge of the last is all that nine parts in ten of the world have trusted to in all ages.'"

That such errors are not prevalent in our time is due in some degree to the free expression of these manly sentiments by men of Heberden's eminence. But our advance is still more owing to the cultivation of morbid anatomy. The facts which it has disclosed first sapped, then overturned, the laborious but unstable structure of empirical experience. The reverence for antiquity in science, so absurd in reason, so unfounded in fact, contradicted alike by wit and observation, is happily gone for ever. The Hippocratists and the Galenists, the Dogmatists and the Empirics of an earlier epoch, have disappeared with the doctorial wig and cane, fit emblems, the one of the dusty learning that it held, the other of the ponderous quarrels which that learning generated.

Dr. Heberden was the author of the following Papers in the Transactions of the College of Physicians:—1. Remarks on the Pump Water of London, and on the Methods of procuring the Purest Water; 2, Observations upon the Ascarides; 3. Of the Night-Blindness, or Nyctalopia; 4. On the Chicken-Pox; 5. The Epidemical Cold, in June and July, 1767; 6. Queries; 7. Of the Hectic Fever; 8. Remarks on the Pulse; 9. Some Account of a Disorder of the Breast; 10. Of the Diseases of the Liver; 11. Of the Nettle Rash; 12. An Account of the noxious Effects of some Fungi; 13. Queries; 14. Case of Angina Pectoris; 15. The Method of preparing the Ginseng Root in China; 16. Of the Measles. The remaining work of Dr. Heberden to be noticed was a posthumous one: Commentarii de Morborum Historia et Curatione.

Leaving this catalogue of his writings, we may inquire, with some degree of curiosity and interest, the character which his practice assumed. A man of great learning, attentive observation, large experience, and considerable natural abilities may be supposed to possess the requisites of a good practitioner. Let Mr. Pettigrew answer for his hero.

"In Dr. Baillie's life, I have endeavoured to account for the apparent inertness of his practice; the same observations will apply to Dr. Heberden. He seems to have had little confidence in remedies—more in Nature herself; and his practice was, therefore, mostly palliative. No analysis of this work can here be given: there is an index to refer to the subjects, which are arranged alphabetically, being preceded by some general remarks on Diet, and the *Ratio Medendi.* The volume concludes with the following reflection, which, if admitted to be correct, must render the prospect of improvement in medicine as next to hopeless. 'The art of healing has scarcely hitherto had any guide but the slow one of experience, and has yet made no illustrious advances by the help of reason; nor will it probably make any, till Providence think fit to bless mankind by sending into the world some superior genius capable of contemplating the animated world with the sagacity shown by Newton in the inanimate, and of discovering that great principle of life, upon which its existence depends, and by which all its functions are governed and directed.'" 18.

The latter passage would indeed, if well founded, tell indifferently, both for reason and for physic. But it must be taken with some degree of limitation, and it seems to be contradicted by another passage of Dr. Heberden's own.

"I please myself," he writes, "with thinking, that the method of teaching the art of healing is becoming every day more conformable to what reason and nature require; that the errors introduced by superstition and false philosophy are gradually retreating; and that medical knowledge, as well as all other dependent upon observation and experience, is continually increasing in the world. The present race of physicians are possessed of several most important rules of practice, utterly unknown to the ablest in former ages, not excepting Hippocrates himself, or even Æsculapius." 6.

The latter sentiments accord, we conceive, more exactly with the truth than the previous ones. The general proposition, that the science of medicine as well as the practical application of it, have not advanced with majestic strides, can be supported only by the hopelessly sceptical or the wholly ignorant. The more limited aspersion, that the progress of medicine has not been due in any material degree to reason is as vulgar a fallacy as ever composed a popular prejudice. Are the operations for aneurysm—the modern doctrines of the arrest of hæmorrhage—the laws and treatment of inflammation of the tissues, mere empirical experience? We cite these instances, because they drop almost spontaneously upon the paper. But even the cases which seem to prove the case and plead the cause of empiricism best, make, when examined, conclusively against it. If accident discovers the utility of a medicine in a given case, it is reason and the experiments which flow from reason which subsequently determine the sphere of its influence and extent of its applicability. To refuse then to reason the lion's share in the present science of medicine, betrays a total want of reflection, or of common sense. We have no patience with this vulgar calumny on physic—begot by an over sceptical mind, and greedily received by those who are unwilling or unable to become thoroughly acquainted with it.

On the whole, it is impossible to read the life of Heberden without arriving at the conclusion that he possessed those qualities calculated to ensure a physician success—mild manners, pure morals, a benevolent heart, a cultivated mind, and a solid, though not a brilliant understanding.

The life of John Cheyne, M.D. Physician General to the Forces in Ireland, a man only lately taken from us, presents some features of interest. John Cheyne was born on the 3rd of February, 1777, at Leith. Many members of his family were of the medical profession. His father practised medicine and surgery, and was a man of great cheerfulness, benevolence, "good sense," and singleness of mind. His family had espoused the cause of tyranny in the person of the Stuarts, and suffered in *pocket* for their pains. After passing four years at the grammar-school of Leith, young Cheyne was, in his tenth year, sent to the High School of Edinburgh, and at once placed under the care of Dr. Adam, the Rector, or Head Master, for whose class he was in no respect prepared. In consequence of this ill-advised step, he is reported to have been, in general, very unhappy while at the school, being unable to keep up with many of his companions, and he often feigned sickness, and submitted to take medicine that he might be kept from school.

Soon after he left the high school, he was placed under the tuition of a clergyman of the Episcopal Church of Scotland, a good scholar, but a foolish and dissipated man: both master and pupil had more relish for talk than for Homer or Virgil; and as he did not exact careful preparation, young Cheyne was seldom prepared, so that, although he went to him daily, for two years, little was added to his stock of Greek and Latin. Thus Cheyne appears to have been a graceless idle lad, and the "good sense" of parents who could not manage him better, must have been of a very questionable character.

assistance of Mr. Candlish, a celebrated grinder of that day, his superficial knowledge of Latin and of medical science was made to answer the end in view, and he passed his examination without difficulty, and obtained a medical degree in June 1795.

On the day after his graduation, (having previously procured a surgical diploma,) he left Edinburgh for Woolwich, the head-quarters of the Royal Regiment of Artillery, to which corps he had been appointed one of the assistant surgeons. He served in various parts of England, till the end of 1797, when he obtained the local rank of surgeon, and accompanied a brigade of horse-artillery, under Lieutenant-Colonel Howorth, to Ireland. With part of that brigade, commanded by Lord Bloomfield, he was present at the actions with the rebels, which took place at Ross, Vinegar Hill, &c., in 1798.

While he was assistant surgeon and surgeon in the Artillery, from 1795 to 1799, his time was spent in shooting, playing billiards, reading such books as the circulating library supplied, and in complete dissipation. He studied nothing but the manners of his superiors, and learnt nothing but ease and propriety of behaviour; happily, however, at last, he became dissatisfied with his prospects, felt anxious to distinguish himself in his profession, and was conscious that unless he made a strenuous effort without delay, he must be content with a subordinate station which his vanity would not have permitted him tamely to occupy: he, therefore, in 1799, left the horse artillery and returned to Scotland, resolved once more to become a medical student. On his return he was appointed to the charge of the Ordnance Hospital in Leith Fort; and he undertook to act as his father's assistant, whose practice, especially among the poor, was very extensive. He selected, for study, the best cases from those which fell to his lot in a division of the business of the day. These cases he journalized; and when he foresaw that a disease would end unfavourably, he took measures to insure a necroscopic examination. At this period he happily formed a friendship with Mr., now Sir Charles Bell, who, then particularly occupied with the study of pathology, was laying the sure foundation of the highest professional eminence. Sir Charles opened most of the bodies which Dr. Cheyne obtained permission to dissect, taught him many things which he might not otherwise have learned, and confirmed his taste for distinction. As an example of diligence in study, Sir Charles could not be excelled; and it was already manifest that he was a man of great originality of mind." 3.

Dr. Cheyne's earlier career would, at first sight, appear exceedingly ill calculated for professional distinction or for professional success. But both, and especially the latter, turn mainly on tact and practical knowledge. Young Cheyne's career was by no means unlikely to teach him something of each. In the society of Artillery officers, he acquired that ease of manner and acquaintance with "the world," useful to him who intends to make "the world" support him; while his early attendance upon *patients* could not fail to render him quick in reading symptoms, as well as prompt in treating them. Knowledge of this kind could not be obliterated even in the shooting-gallery or the billiard-room; and when study added the science that was wanting, the requisites for success were nearly complete.

The object which had now broken on Cheyne's view was pursued with the steadiness of a man of strong sense, confident of his own powers, and determined to turn them to account. He, very probably, felt like Sheridan, after his maiden speech. That was a palpable failure, and most men would have despaired. Not so Sheridan. "By G—d," said he, "it's in me, and it shall come out." If Cheyne did not say this, his conduct proves that he thought something very like it. Observe how he went to work.

"Dr. Cheyne resolved, whenever he should think himself fit for the undertaking, to attempt to establish himself as a physician in a large city, and in the mean while to devote every leisure hour to the necessary preparation. His attention was accordingly directed principally to those diseases which he had the fullest opportunity of studying—to the *diseases of children, acute diseases, and epidemics.* When a well-marked case of disease occurred, or when an epidemic arose, he obtained the best monograph he could on the subject, and attentively compared the information which it contained with the opinions of the most experienced of his professional brethren, whom he had frequent opportunities of meeting; and then he filled up his case-books: thus, by means of observation, reading, and the experience of others, his mind was made up on the most important points of practice, and with decision he acquired a facility of prescription in acute diseases, which proved of great advantage to him, especially in dispensary practice. With respect to chronic diseases, in addition to the assistance he derived from books and observation, he obtained aid from a mass of consultations, many of them written with great care, by the most eminent physicians in Edinburgh, during the middle and end of the eighteenth century, which had been preserved by his granduncle, father, and grandfather Edmondston. Dr. Cheyne endeavoured to become acquainted with the character of those who had obtained the highest rank in the profession of medicine in Edinburgh, to discover the causes of their success; and he ascertained, that although a man might acquire popularity by various means, he could not reckon upon preserving public favour, unless he possessed the respect of his own profession; that if he would effectually guard his own interest, he must, in the first place, attend to the interests of others; hence he was led carefully to study, and liberally to construe, that part of medical ethics which regulates the conduct of physicians towards each other." 4.

We have always been of opinion that success was never attained in our profession without much merit and great exertions of some sort. The merest court physician who ever prescribed a gently medicated dram, neither got nor kept his place without some outlay of cleverness. Many who afterwards saw Cheyne rapidly reaching a large practice, may have wondered at his luck, envied his fortune, and denounced the blindness of public favour. But had they, whom he beat, in the professional race, adopted the same means for securing victory? Did they go upon the course so sagaciously trained as our readers may see that Cheyne was? We will venture to say that the disappointed men of that day had not worked so hard, or so judiciously, for the envied fees, as the man whose "itching palm" clutched and had so tingled for them.

Edinburgh has been long as famous for medical quarrels as for science. Whether it be the natural sharpness of the air, or the diet, or the circumstance of so many doctors being pent in so small a space, certain it is that they are wonderfully given to fight. Pugnacity is almost as striking a characteristic of the surgeons as of the terriers of the town.

During Dr. Cheyne's residence in Scotland, one of these numerous *rows* occurred. Dr. Gregory was the occasion of it, and the "rancour" evinced towards him made such an impression on Cheyne, that he wisely resolved to avoid professional disputes; and to suffer injury rather than attempt to right himself, unless his moral character was likely to be endangered by forbearance. This is an excellent rule, which will be praised by many and acted upon by a few. We proceed with Cheyne's career.

After passing nine years, continues Mr. Pettigrew, in the study of pathology and in the practice of medicine, he resolved upon quitting Scotland, and instituted inquiries in several parts of England without discovering any situation which promised to suit him. He was anxious rather for an opportunity of distinguishing himself than of securing a large income; and with that view he offered his services to the late Dr. Rollo, surgeon-general to the Artillery, who, some years before, wished to establish a school of clinical medicine at Woolwich, for the instruction of the medical officers of the Artillery, then a numerous body. He proposed to give clinical instructions to the junior officers of the establishment, and all he asked in return was the rank and allowances of physician to the forces; but his application came too late—disappointment and disease had quenched Dr. Rollo's zeal for the department over which he had some time before presided with great ability; and Dr. Cheyne never received an answer to his application.

About this time he received a very particular account of the state of the medical community in Dublin, which probably made the deeper impression on him, as it came from one who was not aware of his intended removal from Scotland. He immediately prepared himself for a visit to Dublin, whither he went in the latter end of March, 1809, leaving Mrs. Cheyne in Antrim, with her father, the Rev. Dr. Macartney, vicar of that parish. He soon determined upon remaining in Dublin, in which he found the profession of medicine duly respected. This was chiefly attributable to the eminence of the physicians in that city, during the preceding fifty years: Dr. Smith who was brother to the Archbishop of Dublin, remarkable for his munificence, Sir Nathaniel Barry, whom Mr. Grattan once characterized as the most accomplished gentleman he had ever known, Dr. Quin, Dr. Plunkett, the witty, accomplished and amiable brother of the Lord Chancellor, and Dr. Perceval, distinguished for scientific knowledge, but more so for his philanthropy. Moreover, the memory of Macbride, Cleghorn, and Perceval, was still cherished by many. Dr. Cheyne soon found that the field was extensive, and the labourers liberally rewarded. The physicians then in the confidence of the public were mostly of the school of Cullen—they were possessed of good general information, but relied chiefly on their symptomatology; they had paid but little attention to morbid anatomy. Much of the pure medical practice of Dublin was passing into the hands of the surgeons, who were better acquainted with the nature and tendency of organic lesions. Being well informed on the subject of acute diseases, and having acquired a taste for pathology under Sir Charles Bell, Dr. Cheyne was well prepared for fair competition.

In the latter end of 1809 he took his position as a candidate for public favour in Dublin, where he had passed the Summer; neither expecting nor indeed wishing for rapid advancement. What is easily acquired is little valued and often soon lost. He had a few friends who were much dissatisfied with his apparent apathy, and the obscurity in which he lived; who wished him to go more into company, and even to give entertainments to those who had it in their power to advance his interests; and so much was he urged by his friends to this course, that at last he reluctantly yielded to their importunity; but, as his circumstances did not permit of his providing entertainments with comfort to himself, he refused to repeat the injudicious experiment. True it is, that his friends could derive little encouragement from his apparent progress during the first two years of his sojourn in Dublin:—from the 9th November, 1810, to the 4th of May, 1811, a period of nearly six months, he received only three guineas; but then he felt that prejudices against him were giving way, and that he began to be regarded with good-will by some of the most respectable of his brethren, who, in the latter end of 1811, procured him the situation of physician to the Meath Hospital.

We have extracted these particulars, because we think that they read two important lessons:—first, that a man who aims at success in the practice of medicine, should prepare himself for *possible* opportunities and occasions —must work with no immediate prospect of recompense, perhaps without any clear conception of what he is to do with his knowledge when he gets it; secondly, that, when a man is well prepared, he must not be discouraged at practice coming slowly. We will venture to say that few who have succeeded in our profession have jumped at once into large business. We do not see how, in most instances, that is possible. The confidence of the public is already enjoyed by eminent predecessors. They must secede before the turn of others comes, and such secession implies the lapse of time. Popularity in medical practice got upon the instant is generally the result of whim, of fashion, of some fleeting impulse, which will probably go as it came. If a man, qualified for eminence, makes but three guineas in the first six months, that is no guarantee of success certainly, but experience has proved that it is no indication of failure.

We left Cheyne physician to the Meath Hospital. About this time the directors of the School of Surgery in the Irish College of Surgeons thought it expedient to add to their other professorships, one of the practice of physic. Dr. C.'s attendance on the Meath Hospital procured his election to this office. His lectures at the College of Surgeons, which were very full on the subject of military medicine, were attended by nearly all the surgeons and assistant-surgeons in the garrison, to whom they were free. He delivered five courses of these lectures, and this, with his attendance at the hospital, *and* his private practice, (for practice was now coming,) fully occupied his time.

In October, 1815, he was appointed by the Lord-Lieutenant, one of the physicians to the House of Industry, which lay at the distance of two miles from his house. There he had to visit upwards of seventy patients, in acute diseases, daily; most of these had fever, of which probably eight or ten demanded careful examination. He resigned his professorship at the College of Surgeons, as well as his charge at the Meath Hospital. In 1816, his private practice yielded him £1710.—a not inconsiderable sum for a physician to make in the sixth year after his debut.

Cheyne and Dr. Edward Perceval endeavoured to form a clinical school and a museum of morbid anatomy at the House of Industry. The design proved abortive, but the Dublin Hospital Reports sprang from its ashes.

Upon the death of Dr. Harvey, Physician-general to the Army in Ireland, he applied, without success, for his situation. There were several applicants, whose claims the Lord-Lieutenant found it not easy to adjust, and therefore he escaped from the difficulty by appointing Dr. Perceval, who had not applied for the vacant office; but who, in point of character and professional rank, had a better title to the situation than any of those who were candidates for it. Dr. P. accepted the office, on condition that the Lord-Lieutenant would permit him to have an assistant in the duty of attending the general military hospital. Dr. Cheyne was applied to by Dr. Perceval to assist him in his hospital charge; and, in order to comply with his wishes, Dr. Cheyne thought it necessary to resign his appointment in the House of Industry. Dr. Perceval, however, soon resigned his office, upon which Dr. Cheyne was appointed to succeed him by the Earl Talbot, then Lord-Lieutenant of Ireland. His patent bore the date of Oct. 7, 1820. The situation of physician-general, which was abolished in the end of 1833, was conceived, when Dr. Cheyne obtained it, to confer, on the possessor, the highest medical rank in Ireland; and, as his practice then yielded him about £5,000. (which was its annual average during ten years), he felt that he had fully obtained the object of his ambition.

We have followed Cheyne in his march up hill—we see him at its summit—we are to see him going down. Such are the objects of human desires—sought with avidity—obtained with difficulty—enjoyed with disappointment—and often in themselves the source of irreparable evils. Success in a profession now-a-days has entailed and entails such labour on its possessor, that few who know its real nature can envy it. Success means wealth and eminence, bought with the sacrifice of all healthy recreation, both of body and of mind. The daily toil is relieved only by the nightly anxiety, and, worn by almost uninterrupted exertion, the fortunate man is deprived of most of the social pleasures of life, and debarred from indulgence in its most cherished affections. He acquires property, loses his health, and often leaves the wealth of his industry to be squandered by children whom it demoralises.

Remark Cheyne at the top of his wishes. Let his biographer paint him for us in that happy situation.

His constitution, naturally weak, was always injured by fatigue of body or anxiety of mind. Hence, before long, he was obliged to circumscribe his practice, by refusing to go to a distance from Dublin, or to undertake attendances in the country. He was more generally employed as a consultant than as an attendant physician, and to this line he chiefly adhered. But in the end of the year 1825, when he was about to enter into his forty-ninth year, a period which is often critical to those who are engaged in anxious business, he became affected with a species of nervous fever. In the Autumn of that year, dysentery proved fatal to many of the inhabitants of Dublin. Disappointment often attended the means which were employed for their relief, and a pretty constant depression of his spirits was the consequence of unsatisfactory practice; at the same time, his mind was harassed by anxieties not connected with his profession. He became so weak, that he was unable to dress in the morning till he had had coffee, and when he returned from a day of toil, at seven or eight o'clock in the evening, he was obliged to go to bed, to obtain rest, before he was able to dine. After a struggle of two months, he repaired to England, and recovered some strength, and thought he was again able for business, to which he returned too soon. On his arrival in Dublin, he found one of his professional friends, the father of fifteen children, labouring under a disease, which proved fatal, expecting Dr. Cheyne's return, that he might put himself under his care. The sufferings of this patient proved an incubus on Dr. C.'s spirits, which strangled every cheerful thought. He now began to comprehend the nature of his own illness, namely, that a climacteric disease, what physicians, in former times, termed a nervous atrophy, was forming, which slowly, though surely, was effecting its appointed mission. By relaxing as much from care as he could, sleeping out of town, attending to regularity of meals, getting an experienced medical friend to prescribe for him at the General Military Hospital, and confining himself there to the duties of inspection, the progress of his illness was retarded, and he continued in Dublin till the beginning of 1831, when medical practice at last proved an intolerable burden. "The vigour of the mind decays with that of the body, and not only humour and invention, but even judgment and resolution, change and languish with ill constitution of body and of health."* His sleep was broken and unrefreshing; in the morning he was languid and dispirited; and in the evening, he had a high degree of nervous fever. He resolved therefore to relinquish business, when, to all appearance, ill health alone prevented his retaining it undiminished. Upon his retirement from Dublin, he was addressed by the physicians, the surgeons, and the apothecaries, of the Irish metropolis. The addresses expressed, in the warmest terms, the sentiments of esteem they entertained for his private virtues, and the respect they felt for his professional character—deeply deplored the necessity of his retirement, and ardently hoped for his recovery—hopes, never to be realized.

[illegible]. Cheyne sought a quiet English village, and found it in Newport Pagnell, in Buckinghamshire, where he had purchased an estate in 1829. But the retirement of a physician is seldom absolute. It is one distinctive feature of our philanthropic profession, that the ability to exercise it with benefit to our fellow-creatures ends only with the total prostration of our powers. When the acquisition of wealth has ceased to please or to be requisite, we have it in our power to assuage human miseries unfettered by the considerations of self-interest, and unsuspected of its bias. How much must the decline of life be sweetened by the gratuitous employment of our medical skill. The eminent physician, occupying his sickness or his age in ministering to the bodily afflictions of the poor, is surely a spectacle worthy of the admiration of gods and men: and such a spectacle was presented by Cheyne.

Three mornings in the week, he went to a neighbouring cottage, and saw the sick villagers, giving them advice, and dispensing medicines, which were prepared in his family; thus, many an attack of illness was nipped in the bud, and much suffering was lessened. On a fourth morning, the sick came to him from distant parts of the country, for whom he prescribed: and, as there was no physician within twelve miles of the post-town nearest his house, he was occasionally consulted by some of the more respectable families in the neighbourhood. Surely a profession which admits of this has some claims on the respect of men.

Cheyne felt that he owed a duty to that profession, as well as to the general interests of humanity. Wealth and distinction acquired in a profession are to be regarded in some degree as trusts held for its benefit. The man who by it has obtained those objects of human ambition, must feel that he owes something to a science which has enabled him to turn his talents to so good account. No man should let the fruits of his experience rot with

* Sir William Temple.

him. They are an offering which he is bound to make upon the shrine of knowledge, a votive tablet hung, like the records of the cases in the temple of Esculapius, to signalise his own escape from disasters, and to thank the Gods for their protection.

Cheyne entertained this feeling. Something may, perhaps, be laid to the score of a mind;—

which, working out its way,
Fretted the pigmy body to decay, uneasy at the forced inaction of the body, and carving out for itself a sphere of action. Yet, allowing all that the most peevish cynicism could justly ask, there must have been a vein of noble feeling in the breast of Cheyne. He wished, his biographer tells us, to prove that he still retained an interest in his profession, even after it had ceased to yield him emolument; and, therefore, he gladly undertook to write some articles for the Cyclopædia of Practical Medicine, in compliance with the request of Dr. Tweedie, one of the Editors of that work. He was thus again led to the use of his pen, and began to extend his inquiries to other subjects, recollecting and recording facts and reasonings, which, in the hurry of business, he had almost let slip. But Fortune had bestowed her last smile on Cheyne, and his closing years were to be darkened by deprivations most keenly felt by those who have led a life of intellectual activity. A cataract formed in his right eye in the beginning of 1833, which deprived him of the use of that organ. His left eye became dim, and his strength altogether, so much exhausted, that he was obliged to relinquish any further pursuit in his profession. His frame now gradually verged towards decay; four months prior to his decease, a mortification of one of his feet took place, and he sank on the 31st of January 1836.

The opinion entertained of Cheyne by his contemporaries, may be gathered from a short biographical sketch of him, contained in the Dublin Journal.

"To speak of Dr. Cheyne's writings, and of the estimation in which they are held, would be superfluous. Whether viewed with reference to the elegance of their style, or the sound practical precepts and improvements which they inculcate, they hold a foremost rank among the medical works of the day. No man ever maintained, in the circle in which he practised, more respect and confidence from his professional brethren, or a higher character with the public as a skilful physician. In consultation, he displayed a penetration in the diagnosis of disease, and a readiness and sincerity in the communication of his experience in similar cases, which never failed to secure the confidence of the practitioner, at whose recommendation he had been called to the attendance. He had, moreover, the enviable quality of observing the most honourable conduct toward the gentleman in attendance with him, without a compromise of the duty which he owed to the invalid: he directed the treatment, without arrogating to himself any merit for its success; and assisted the efforts of his junior without lessening towards him the confidence of his patient. Dr. Cheyne's punctuality to appointments was another feature in his character, which rendered professional intercourse with him peculiarly satisfactory; and even in the days of his most varied and extensive practice, he treated the youngest member of the profession with the same polite consideration, in this particular, as the oldest."

"His exterior deportment bore the appearance of indifference to the afflictions which were every day presented to him; but his inward feelings on those occasions were at variance with the impressions left by those visits. His mind was frequently disturbed, and his spirits depressed, by the scenes of bodily and mental suffering, which the practice of his profession incessantly brought under his notice; and his sympathies, instead of being blunted by the habitude of meeting with such objects of compassion, were rendered more acute by the repetition. Indeed, to this source may be traced the seeds of the malady under which his constitution, otherwise a good one, finally broke down." 10.

Mr. Pettigrew adds:—

"Dr. Tweedie has confirmed this statement to me; and, as an evidence of Dr. Cheyne's sensitiveness, says that he once told him, he had that day seen thirteen patients, all of whom were moribund, and that he therefore did not expect any of them to be alive on the morrow. This, he said, was too much for him—he was unable to bear it, and he determined to fly from such scenes of hopeless and helpless affliction." 11.

It would not be right to close this notice without an enumeration of Cheyne's writings. The following are the heads of the Essays or the Papers that he published:—

1. An Essay on Cynanche Trachealis.

2. On the Bowel Complaints more immediately connected with the Biliary Secretion, and particularly of Atrophia Ablactatorum.

3. On Hydrocephalus Acutus, or Dropsy of the Brain.

4. On the Pathology of the Membrane of the Larynx and Bronchia.

5. Cases of Apoplexy and Lethargy: with Observations upon the Comatose Diseases.

In the Dublin Hospital Reports, Cheyne published the following ten papers:—

6. Report of the Hardwicke Fever Hospital, for the year ending on the 31st March, 1817; A Case of Melæna, with Observations on the Alternate Excess of Morbid Action in the Mucous and Serous Membranes; Of Jaundice, unaccompanied by any discoverable disease of the Liver, or turgescence or obstruction of the Biliary Ducts; On the Virtues of James's Powder in the Apoplectic Diathesis; Report of the Hardwicke Fever Hospital, for the year ending on the 31st March, 1818, including a brief account of an Epidemic Fever in Dublin; Case of Apoplexy, in which the fleshy part of the Heart was converted into fat; Report of the Whitworth Hospital, containing an account of Dysentery, as it appeared in Dublin in the latter end of 1818; with a brief account of that Disease, as it appeared at Limerick in 1821; Medical Report on the Feigned Diseases of Soldiers; Cases of a Fatal Erethism of the Stomach, with Observations; On Small and Frequently-repeated Bleedings in Hæmoptysis and Incipient Phthisis.

Cheyne contributed two Papers to the Edinburgh Medical and Surgical Journal.

1. A case of Bronchial Polypus.

2. Observations on the Effects of Purgative Medicines.

There are five Articles from his pen in the Cyclopædia of Practical Medicine.

1. Croup.
2. Epilepsy.
3. Epidemic Gastric Fever.
4. Laryngitis.
5. Wakefulness.

Cheyne's life appears to us to derive its main interest from the lesson it reads on the mode of obtaining success in the profession. Without very brilliant talents—without very powerful friends—without any striking piece of good fortune, Cheyne reached eminence and acquired a large income. That he did so, was clearly owing to his having devoted his attention to *practical* studies, and directed his aims to *practical* attainments. He adopted those means which plain good sense and a clear head would indicate as likely to answer, and they did answer.

